# Identification and Verification of Hub Mitochondrial Dysfunction Genes in Osteoarthritis Based on Bioinformatics Analysis

**DOI:** 10.1155/2024/6822664

**Published:** 2024-01-23

**Authors:** Hui Niu, Xingxing Deng, Qian Zhang, Yijun Zhao, Jinfeng Wen, Wenyu Li, Huan Liu, Xiong Guo, Cuiyan Wu

**Affiliations:** School of Public Health, Xi'an Jiaotong University Health Science Center, Key Laboratory of Trace Elements and Endemic Diseases, National Health Commission of the People's Republic of China, Xi'an 710061, Shaanxi, China

## Abstract

**Objective:**

Age-related mitochondrial dysfunction and associated oxidative stress may contribute to the development of osteoarthritis. The aim of this study was to identify hub genes associated with mitochondrial dysfunction in osteoarthritis (OA) patients, helping predict the risk of OA, and revealing the mechanism of OA progression.

**Methods:**

OA expression data and mitochondrial dysfunction genes were downloaded from GEO (GSE55235, GSE82107, and GSE114007) and GeneCard databases. The differentially expressed mitochondrial dysfunction genes (DEMDFGs) between OA and control samples were screened. Gene ontology (GO) and Kyoto encyclopedia of genes and genomes pathways were analyzed for DEMDFGs. The hub genes were determined by WGCNA and LASSO regression analysis. ROC curves manifested the diagnostic efficacy of each hub gene. A nomogram model was constructed and validated to predict OA risk. The expression of hub genes in OA and normal chondrocytes was verified by external datasets, qRT-PCR and western blotting.

**Results:**

A total of 31 DEMDFGs were identified, with 15 genes upregulated and 16 genes downregulated. GO functional enrichment analysis revealed that DEMDFGs were enriched in biological processes related to energy metabolism and cellular respiration. By employing weighted gene coexpression network analysis, we identified four distinct coexpression modules, among which the blue module exhibited the strongest correlation with OA. The intersection between DEMDFGs and this module yielded eight candidate genes. After LASSO analysis of the data, four hub genes (ACADL, CYBA, SLC19A2, and UCP2) were identified as potential biomarkers for OA. The expression levels of these four genes were externally validated in the GSE114007 dataset. And the biologically differential expression of these four genes has been verified in OA and normal chondrocytes. Moreover, the four hub genes had good sensitivity and specificity by ROC curve analysis, and the risk model constructed with these four genes showed promising performance. In conclusion, our study may provide novel mitochondrial dysfunction hub genes with potential clinical applications for understanding the pathology, diagnosis, and treatment of OA.

## 1. Introduction

Osteoarthritis (OA) is a prevalent chronic and degenerative joint disease that primarily affects weight-bearing joints (knee and hip) and hand joints. Its incidence and prevalence are increasing annually with global aging and the increase of risk factors for OA, which has affected the life of one-third of 65-year-olds and become a major cause of disability in the elderly, imposing a heavy burden on the patient's family and society [1, 2]. OA is considered an age-related disease, and gender, overweight or obesity, knee injury, and occupation can all increase the risk of developing osteoarthritis [2]. It is characterized by chondrocyte reduction and extracellular matrix degradation as the main pathological changes [3], which in turn affects the synovial membrane and the subchondral bone of the joint, with the formation of osteophyte, leading to a series of joint symptoms and signs, such as joint pain, stiffness, reduced mobility, which in severe cases can lead to decreased physical function, impaired sleep, depression, and even disability, seriously affecting the quality of life of patients [4]. It has been shown that OA also affects young adults, with profound effects in terms of psychological health and work capacity [5]. However, the pathogenesis of OA has not been fully elucidated, and the early prediction and specific therapy of OA remain a challenging problem. Therefore, biomarker studies that contribute to early prediction are particularly important and could facilitate individualized treatment.

Mitochondria are essential organelles that coordinate various metabolic processes in cells and play important contributions to cellular bioenergetics and apoptosis [6]. Age-related mitochondrial dysfunction and oxidative stress may contribute to the development of osteoarthritis [7]. Mitochondrial dysfunction is mainly characterized by reduced adenosine triphosphate (ATP) production, increased ROS production, imbalance in calcium homeostasis and mitochondrial DNA, leading to chondrocyte damage [8]. ATP production in chondrocytes is heavily dependent on oxidative phosphorylated (OXPHOS). Mitochondrial dysfunction can lead to reduced activity of respiratory chain complexes resulting in reduced OXPHOS in OA chondrocytes, disrupting the balance between glycolysis and OXPHOS, thereby greatly reducing ATP production and damaging chondrocytes, while inducing the release of interleukin-1*β* from chondrocytes leading to inflammation [9, 10]. ROS accumulation and mtDNA damage can activate the NF-kB pathway to regulate inflammatory factors. Studies have shown that lower levels of ROS are beneficial in maintaining chondrocyte homeostasis, while excessive production of ROS, including hydrogen peroxide and nitric oxide, plays a key role in the pathogenesis of OA [8]. Mitochondrial uptake of calcium ions is used to regulate their activity, stimulate ATP synthesis and regulate mitochondrial enzymes involved in the tricarboxylic acid (TCA) process. High concentrations of calcium ions lead to cell death, while low concentrations disrupt cellular energy metabolism [11]. Mitochondrial dysfunction causes programed cell death, in which mitochondrial autophagy plays an important role. Proper autophagy removes damaged mitochondria having a protective effect on OA cartilage and chondrocytes, while excessive autophagy reduces a significant number of important cellular components (CCs) resulting in cytotoxic effects [12]. Mitochondrial dysfunction also promotes the release of pro-inflammatory mediators and the accumulation of inflammatory cells, thereby inducing an inflammatory response in synovial cells [13, 14]. Inflammatory synovium that occurs in the early stages of OA likewise produces the pro-inflammatory mediator nitric oxide, cytokines, and prostaglandin E (2), which exacerbate cartilage changes by altering the balance of cartilage matrix degradation and repair. The chondrocyte changes in turn amplify synovial inflammation, creating a vicious cycle [15]. In conclusion, dysfunctional mitochondria could regulate multiple related cell proliferation, differentiation, and death by affecting multiple networks of signaling molecules, which in turn promote the development of OA.

The study screens for mitochondrial dysfunction genes that are significantly differentially expressed in OA patients compared to the normal group, providing a novel basis for the pathological, diagnosis, and treatment clue of OA with great potential for application in clinical practice.

## 2. Materials and Methods

### 2.1. Data Acquisition and Processing

**Figure 1:**
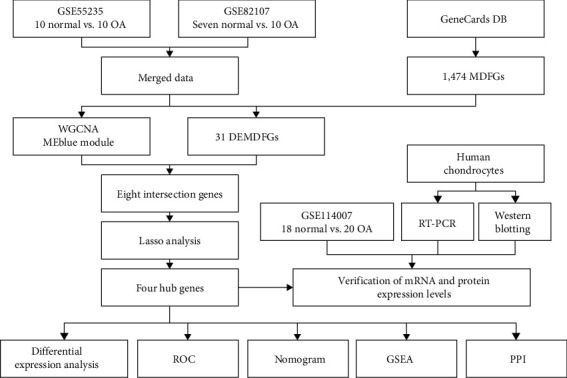
Flowchart of the analysis process. OA, osteoarthritis; DB, database; MDFGs, mitochondrial dysfunction genes; WGCNA, weighted gene coexpression network analysis; DEMDFGs, differentially expressed mitochondrial dysfunction genes; ROC, receiver operating characteristic; GSEA, gene set enrichment analysis; and PPI, protein–protein interaction networks.

The workflow of this study is illustrated in Figure 1. Three OA gene expression microarray datasets (GSE55235, GSE82107, and GSE114007) were obtained from the NCBI Gene Expression Omnibus (GEO; https://www.ncbi.nlm.nih.gov/geo/). The GSE55235 dataset is based on the GPL96 platform (HG-U133A) Affymetrix Human Genome U133A Array and includes 10 normal and 10 OA samples; while the GSE82107 dataset is based on the GPL570 platform (HG-U133_Plus_2) Affymetrix Human Genome U133 Plus 2.0 Array and contains seven normal and 10 OA samples. GSE114007 is based on the GPL18573 platform Illumina NextSeq 500 (*Homo sapiens*) and consists of 18 normal and 20 OA samples. Bioconductor's surrogate variable analysis package was employed to eliminate batch effects and merge the GSE55235 and GSE82107 datasets [16]. In addition, 1,474 mitochondrial dysfunction genes (MDFGs) were collected from the GeneCards (https://www.genecards.org/) database according to the relevance score >5 [17].

### 2.2. Differentially Expressed MDFGs Screening

The “limma” package was applied to screen differentially expressed MDFGs (DEMDFGs) and adjusted *P*-value < 0.05 and |log2 fold change (FC)| > 1 were used as the criteria for selecting DEMDFGs [18]. Heat map and volcano map of DEMDFGs were obtained by using “heatmap” and “ggplot2” packages, respectively. The DEMDFGs were analyzed using gene ontology (GO) and Kyoto encyclopedia of genes and genomes (KEGG) pathway enrichment network online analysis tool Metascape (http://metascape.org).

### 2.3. Weighted Gene Coexpression Network Analysis (WGCNA) and Hub MDFGs Identification

Weighted gene coexpression network analysis (WGCNA) analysis was carried out on the merged dataset to explore gene interactions and identify coexpression of genes and modules [19]. Genes with standard deviation (SD) > 0.75 between OA and normal samples were selected for analysis, and outlier samples were removed. Soft thresholds (*β*) were selected and validated using the “PickSoftTreshold” soft threshold function, in which the networkType parameter was selected as “unsigned”. The coexpression modules were formed from genes with comparable expression characteristics. For modules related to clinical attributes, module affiliation (MM) and gene significance (GS) were calculated. Potential hub genes related to OA were evaluated using the MM > 0.8 and GS > 0.5 thresholds. The same genes between DEMDFGs and potential hub genes in significant modules were obtained as common genes (CGs). Further, to identify the hub MDFGs for OA, least absolute shrinkage and selection operator (LASSO) regression analysis was conducted by using the “glmnet” package for CGs.

### 2.4. Diagnostic Value of Hub MDFGs and Construction of Risk Model

Differential analysis was performed for each hub MDFG to identify its differential expression in OA samples and controls, and ROC curves were plotted to examine the diagnostic efficacy of each hub MDFG gene for OA. A nomogram model was established to predict the risk of OA by using the “rms” package. Then, calibration curve was used to assess the predictive power of the nomogram model. The R package “ROCR” was employed to perform ROC analysis and calculate the area under the curve (AUC).

### 2.5. Pathway Enrichment Analysis and PPI Network Construction of Hub MDFGs

Gene set enrichment analysis (GSEA) was used to perform KEGG enrichment analysis [20]. |NES| > 1 and FDR < 0.25 were considered significantly enriched. To further analyze the biology functions of the hub MDFGs, the GeneMANIA (http://www.genemania.org) online tool was applied to constructing PPI network, which generated hypotheses about gene function, analyzed gene lists, and prioritizes genes for functional analysis [21].

### 2.6. Validation of Hub MDFGs in OA

The GSE114007 dataset was introduced as an external validation dataset to verify the expression level and predictive power of hub MDFGs in OA cartilage tissue.

### 2.7. Chondrocytes Samples

The study was approved by the Ethics Committee of Xi'an Jiaotong University (Number: 2020.1043). Articular cartilage samples were obtained from three patients with OA who underwent knee replacement surgery and three donors without joint disease. All cartilage samples were extracted and processed in accordance with the Declaration of Helsinki. The cartilage tissue was cut into small tissue pieces by scalpel, and washed with PBS for three times, and 0.25% (m/v) trypsin (HyClone, USA) was added to continue cutting the small tissue pieces. Afterward, type II collagenase (Gibco, USA) was added overnight at 37°C in a shaker. The chondrocytes were washed three times with DMEM/F12 medium (HyClone, USA), and the resulting precipitate after centrifugation was chondrocytes. The chondrocytes were cultured in DMEM/F12 medium (HyClone, USA) containing 10% fetal bovine serum (Gbico, USA) and 1% penicillin and streptomycin (Sigma, USA) at 37°C with 5% CO_2_ concentration, and the culture medium was changed every 2 days and the nonadherent cells were removed.

### 2.8. qRT-PCR Validates mRNA ExpRession of the Hub MDFGs in OA

The chondrocytes from three OA patients and three healthy donors were collected for qRT-PCR to confirm whether the expression of the screened hub gene in OA patients was consistent with the results of the datasets. Total RNA was extracted from chondrocytes using SteadyPure Universal RNA Extraction Kit Ⅱ (code no. AG21022, Accurate) according to the manufacturer's protocol. The total RNA was reverse transcribed into cDNA with StarScript Ⅲ RT MasterMix (GenStar, China). qRT-PCR was performed in three technical replicates using CFX96Real-Time PCR Detection System (Bio-Rad) with SYBR® Green Premix Pro Taq HS qPCR Kit (code no. AG11701; Accurate). GAPDH was used as an internal control. The gene expression levels were estimated using the 2^−*ΔΔ*Ct^ methods. The *t*-test was used for the statistical analysis with the significant difference of *P* < 0.05. The primers used for qRT-PCR experiment are listed in Table 1.

### 2.9. Western Blotting

Chondrocytes were lysed using RIPA lysis buffer (Epizyme, China) containing phosphatase and protease inhibitors. Total proteins were separated by SDS polyacrylamide gel electrophoresis (PAGE) and then transferred to an activated polyvinylidene difluoride (PVDF) membrane. After blocking with 5% skim milk, the PVDF membranes were incubated with primary antibody at 4°C overnight. The primary antibodies were ACADL (1 : 1,000, Immunoway, YT6498), CYBA (1 : 1,000, Immunoway, YN1733), SLC19A2 (1 : 1,000, Bioss, bs-10738R), UCP2 (1 : 1,000, Immunoway, YT4813) and GAPDH (1 : 10,000, Proteintech, 10494-1-AP). The blotting membrane was then incubated with the corresponding goat anti-rabbit IgG secondary antibody (1 : 5,000, Proteintech, SA00001-2) for 2 hr at room temperature. The surface of the blotting membrane was then covered with ECL chemiluminescent reagent (Epizyme, China), and the protein bands were detected by a chemiluminescent system (Bio-Rad, USA). Furthermore, the blot images were assessed for normalization and quantification using ImageJ.

## 3. Results

### 3.1. DEMDFGs Identification

**Figure 2:**
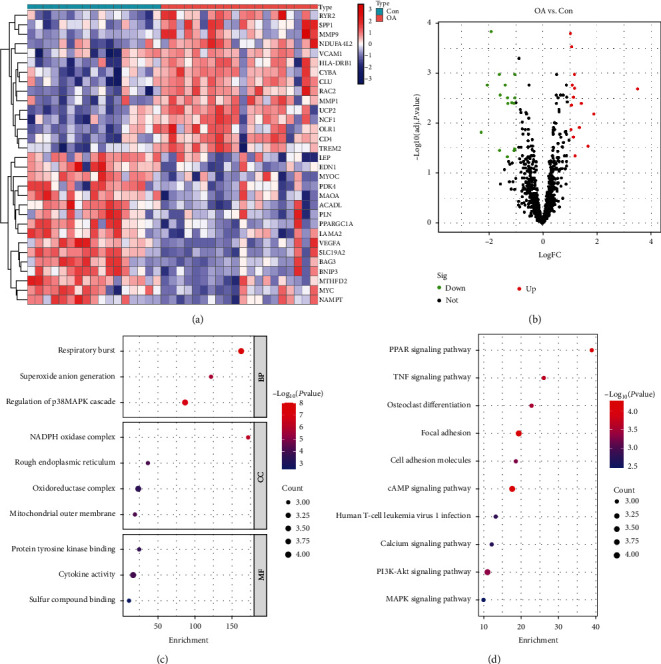
Heat map and volcano plot for the DEMDFGs identified from the integrated dataset. (a) Each row of the heatmap represents one DEMDFGs, and each column represents one sample. (b) The red plot points represent the upregulate genes, and the green plot points represent the downregulate genes. (c) GO analysis of DEMDFGs. (d) KEGG analysis of DEMDFGs. OA, osteoarthritis; con, control.

After intersecting the collected MDFGs with the merged data for taking intersection, 1,133 genes were identified. A total of 31 genes were differentially expressed, of which 15 genes were upregulated and 16 genes were downregulated (Figures 2(a) and 2(b)). Then, GO enrichment was used to analyze the biological processes, CCs, and molecular functions (MFs) of these DEMDFGs (Figure 2(c)). For GO analysis, the enriched items mainly participated in respiratory burst, superoxide anion generation, NADPH oxidase complex, protein tyrosine kinase binding, etc. KEGG analysis was used to further research the pathway of those genes (Figure 2(d)). The results showed that DEMDFGs participated in the pathways in PI3K-Akt signaling pathway, PPAR signaling pathway, TNF signaling pathway, etc.

### 3.2. Hub MDFGs Identification

**Figure 3:**
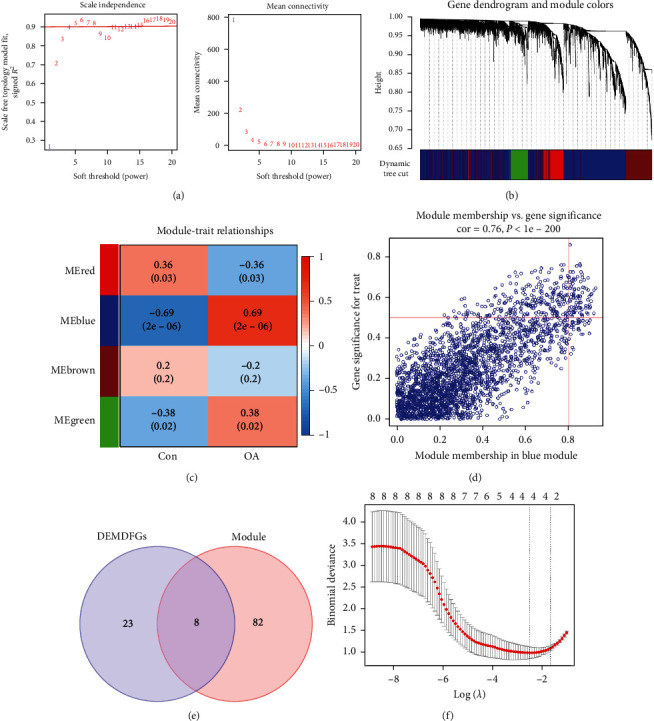
Weighted gene coexpression network analysis and LASSO regression analysis screening hub MDFGs: (a) determine the best soft threshold; (b) gene coexpression modules, each color represents a coexpression module; (c) Heat map of the association between modules and OA; (d) correlation plot between MM and GS of genes contained in the blue module; (e) Venn diagram showing overlapping genes between the DEMDFGs and the candidate hub genes in the blue module; and (f) LASSO regression analysis.

The genes were ranked according to the SD from largest to smallest and those with SD > 0.75 were selected for analysis. The power parameters in the range of 1–20 were filtered in the WGCNA package. In our study, a power of *β* = 4 (scale-free *R*^2^ = 0.9) was chosen as the soft threshold to ensure a scale-free network (Figures 3(a) and 3(b)). A total of four modules were obtained in which the genes have similar coexpression characteristics. The module signature gene in the blue module (*r* = 0.69; *P*=2e − 06) was significantly correlated with OA (Figure 3(c)). The significant positive correlations between gene's MM and GS were obtained in the blue module (cor = 0.76, *P* < 1e − 200), which included 90 candidate hub genes (Figure 3(d)). The screened DEMDFGs were intersected with the candidate hub genes in the blue module to obtain eight hub genes (Figure 3(e)). Finally, a total of four hub genes were obtained by LASSO regression analysis, which are ACADL, CYBA, SLC19A2, and UCP2 (Figure 3(f)).

### 3.3. Diagnostic Value of Hub MDFGs and Risk Model

**Figure 4:**
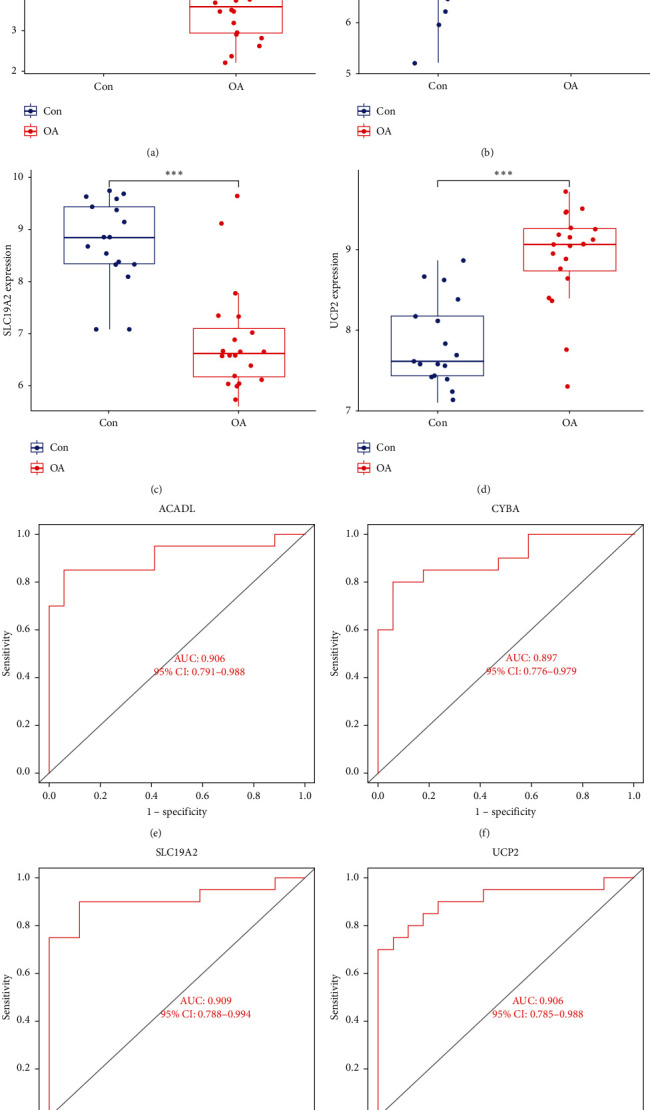
Expression levels and ROC curves of hub MDFGs. (a–d) Box plots of the difference in expression levels of hub MDFGs between OA and control groups. (e–h) ROC curves manifested the predictive efficacy of each hub gene.  ^*∗∗∗*^*P* < 0.001. AUC, area under the curve.

The expression of the four hub MDFGs was analyzed in the OA and control groups. In the OA group, CYBA and UCP2 were upregulated, while ACADL and SLC19A2 were downregulated (Figure 4(a)–4(d)). Further calculation of the diagnostic potency of each gene for OA showed that the AUC of ACADL, CYBA, SLC19A2, and UCP2 were 0.906, 0.897, 0.909, and 0.906, respectively (Figure 4(e)–4(h)). The AUC values for all four central genes were greater than 0.85, indicating that these genes have excellent specificity and sensitivity for diagnosis of OA.

**Figure 5:**
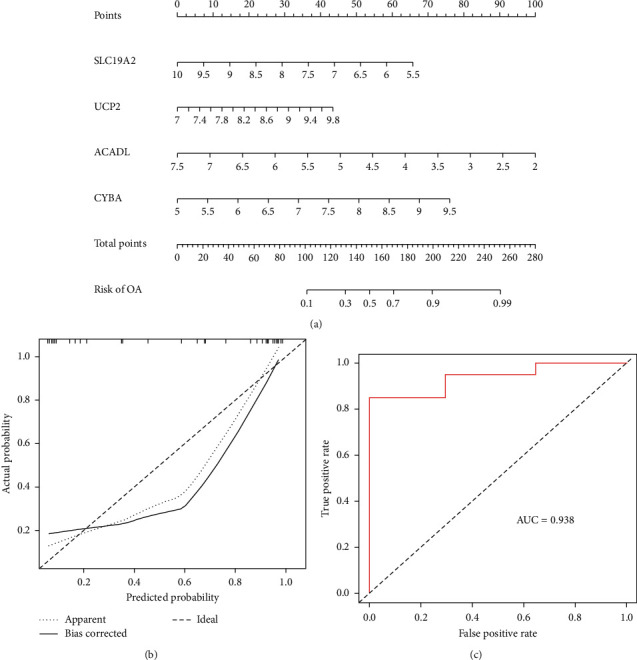
Construction and validation of a nomogram model for predicting OA risk. (a) Nomogram to predict the risk of OA occurrence. (b) The calibration curve evaluates the predictive power of the nomogram model. (c) ROC curves analysis evaluates the accuracy of the nomogram model.

To further confirm the ability of these four genes to predict OA risk, a nomogram model for OA risk based on the four hub MDFGs was established (Figure 5(a)). The calibration curve showed that the error between the actual OA risk and the predicted risk was modest (Figure 5(b)), indicating that the nomogram model predicts the risk of OA with excellent accuracy. The ROC analysis of the model was further performed (Figure 5(c)), and the AUC for this model was 0.938. These results suggested in some respects that these four MDFGs may have a crucial role in the development of OA.

### 3.4. Pathway Enrichment Analysis of Hub MDFGs

**Figure 6:**
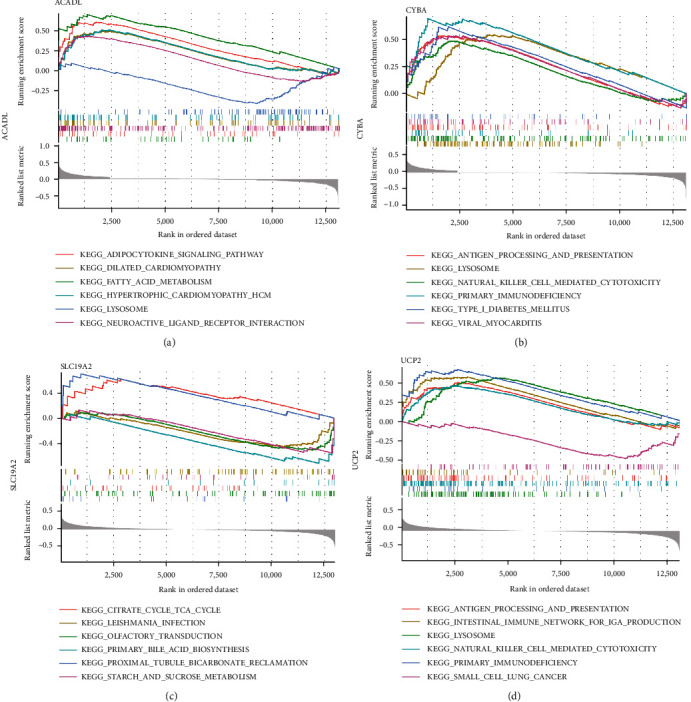
Significant pathways implicated with ACADL, CYBA, SLC19A2, and UCP2: (a) KEGG analysis of ACADL; (b) KEGG analysis of CYBA; (c) KEGG analysis of SLC19A2; and (d) KEGG analysis of UCP2.

Based on GSEA, the four hub MDFGs associated pathways were further analyzed. ACADL is mainly enriched in KEGG fatty acid metabolism, adipocytokine signaling pathway, and lysosome (Figure 6(a)). CYBA major involved in KEGG natural killer cell-mediated cytotoxicity, primary immunodeficiency, antigen processing, and presentation (Figure 6(b)). For SLC19A2, citrate cycle TCA cycle, primary bile acid biosynthesis were mostly enriched pathways (Figure 6(c)). The main enrichment pathways of UCP2 were natural killer cell-mediated cytotoxicity, intestinal immune network for IGA production, primary immunodeficiency, antigen processing, and presentation (Figure 6(d)).

### 3.5. Protein–Protein Interaction Network Analysis

**Figure 7:**
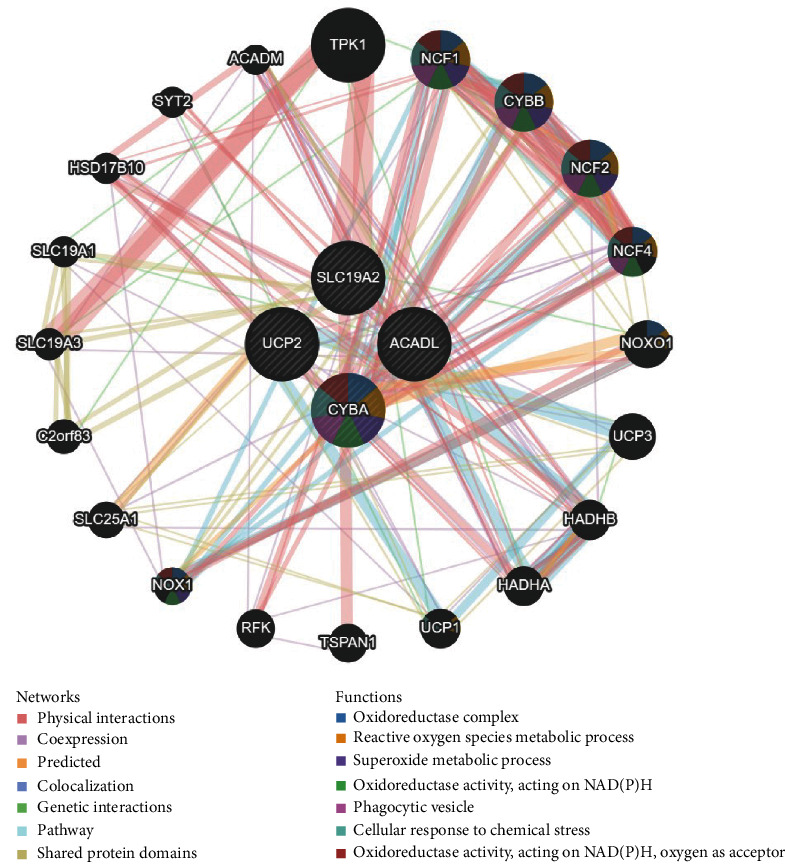
PPI network and functional analysis of four hub MDFGs.

The protein–protein interaction network based on the GeneMANIA database was structured, in which four hub MDFGs and their interconnections were found (Figure 7). The four hub genes were at the central node, surrounded by 20 genes that are notably correlated with them. Distinct colors of the network edge indicated the bioinformatics methods applied: physical interactions, coexpression, predicted, colocalization, pathway, genetic interactions, and shared protein domains. The results showed that the gene CYBA was functioned in oxidoreductase activity, oxidoreductase complex, superoxide metabolic process, reactive oxygen species metabolic process, etc.

### 3.6. Validation of Hub MDFGs in OA

**Figure 8:**
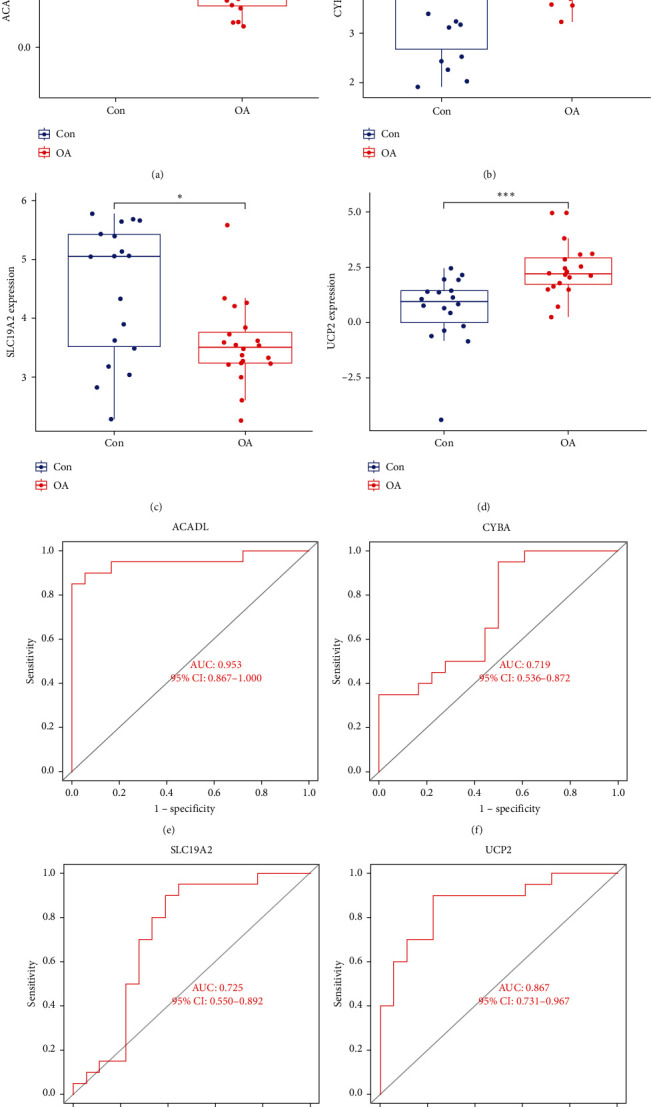
Validation of hub MDFGs with GSE114007 dataset. (a–d) Box plots of the difference in expression levels of hub MDFGs between OA and control groups. (e–h) ROC curves manifested the predictive efficacy of each hub gene. AUC, area under the curve. ( ^*∗*^*P* < 0.05,  ^*∗∗∗*^*P* <  0.001).

To further determine the expression level and predictive power of the screened hub MDFGs, we performed validation in another dataset, GSE114007. The results showed that ACADL and SLC19A2 were lowly expressed in OA samples, while CYBA and UCP2 were highly expressed in OA samples, which is consistent with our previous screening results (Figure 8(a)–8(d)). Meanwhile, the four hub MDFGs also showed relatively well predictive power (Figure 8(e)–8(h)).

### 3.7. mRNA and Protein Expression Levels of Hub Genes in OA and Control Chondrocytes

**Figure 9:**
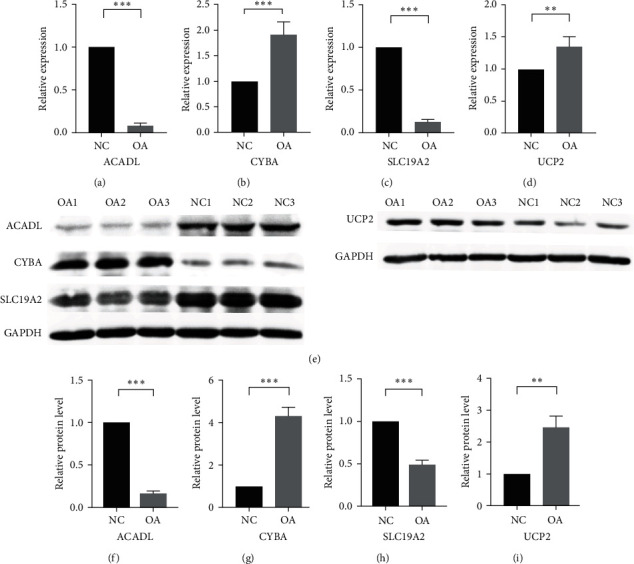
qRT-PCR and WB validation of the hub genes between OA and control chondrocytes. (a–d) The relative mRNA expression levels of each gene were calculated using 2^−*ΔΔ*Ct^ methods. (e–i) WB assay and quantitative analysis showed ACADL, CYBA, SLC19A2, and UCP2 protein levels in chondrocytes. ( ^*∗∗*^*P* < 0.01,  ^*∗∗∗*^*P* < 0.001).

The relative expression of ACADL, CYBA, SLC19A2, and UCP2 in OA and normal control chondrocytes were detected by qRT-PCR and western blotting (WB). Compared to the control chondrocytes, the mRNA expression of ACADL and SLC19A2 was significantly downregulated, while the mRNA expression of CYBA and UCP2 was upregulated in OA chondrocytes (Figure 9(a)–9(d)). The WB assay results revealed that the OA group exhibited increased protein expression levels of CYBA and UCP2, while decreased protein expression levels of ACADL and SLC19A2 in comparison to the normal control group (Figure 9(e)–9(i)). The mRNA and protein expression of ACADL, CYBA, SLC19A2, and UCP2 were in accordance with the previous data analysis.

## 4. Discussion

Mitochondria perform a crucial function in maintaining chondrocyte homeostasis. In the joint environment and inflammatory conditions of osteoarthritis, the occurrence of mitochondrial dysfunction further accelerates the disease process. In the present study, we systematically investigated the differential expression of genes associated with mitochondrial dysfunction in OA and normal subjects. A total of 31 DEMDFGs were differentially expressed, of which 15 genes were upregulated and 16 genes were downregulated. Finally, we have identified four hub genes (ACADL, CYBA, SLC19A2, and UCP2) which had relatively good sensitivity and specificity. The expression of the hub genes was further verified in chondrocytes from OA patients and normal control.

The role of the four hub genes of mitochondrial dysfunction in OA is primarily manifested as reduced ATP production, increased oxidative stress, and lipid metabolism disorder. ROS is produced by mitochondrial and plasma membrane-localized NADPH oxidase and plays a pivotal role in the initiation and progression of OA. ROS accumulation in chondrocytes could lead to oxidative damage and even apoptosis. In addition, there is a correlation between increased oxidative stress and the induction of cartilage senescence, which may lead to osteoarthritis [7]. Increased CYBA expression may accelerate the progression of OA by producing excess ROS and causing cell death. Fortunately, increased UCP2 expression in OA patients is modulated by negative feedback to alleviate oxidative stress damage. In OA, abnormal chondrocyte metabolism caused by inflammatory factors may further promote the development of cartilage degeneration and disease. When exposed to environmental stimuli, such as an inflammatory environment or external stress, chondrocytes may adapt to changes in the microenvironment by altering metabolic pathways [22]. In addition to the influencing factors of abnormal lipid metabolism in chondrocytes, some clinical studies have found that lipid metabolism in blood is also connected with the development of OA [23, 24]. Articular chondrocytes, unlike most other cells, are characterized by large lipid deposits. In OA patients, the decreased expression of ACADL could lead to the dysfunction of lipid metabolism in cells. The accumulation of excess lipids in cells produces lipid toxicity, resulting in cell dysfunction and/or cell death.

ACADL, namely long-chain acyl-coenzyme A dehydrogenase, belongs to the acyl-coenzyme A dehydrogenase superfamily, which is required for catalyzing the first step of long-chain lipid acyl-coenzyme A *β*-oxidation [25]. ACADL deficiency causes hepatic and cardiac lipidosis, hypoglycemia, and impaired fatty acid oxidation in mice. With the decline of mitochondrial function and the decrease of mitochondrial content, it further leads to increased lipid accumulation [26]. The level of cartilage lipid accumulation was positively correlated with the severity of osteoarthritis [27]. It has been shown that chondrocytes from patients with osteoarthritis exhibit incomplete *β*-oxidation of fatty acids compared to healthy individuals and are accompanied by mitochondrial damage in cell populations, which may be associated with the downregulation of ACADL expression [28]. Additionally, the dysregulation of lipid metabolism may be responsible for the increase of ROS in senescent cells [29]. At the same time, impaired fatty acid oxidation affects the energy supply of synovial cells, leading to an increase in cellular stress and inflammatory responses, resulting in the release of inflammatory mediators and cytokines, and leading to the development and progression of synovial inflammation [30].

The CYBA gene is used to encode the p22 (phox) protein, which is a key component in the production of superoxide nicotinamide adenine dinucleotide phosphate (NADPH) oxidases (NOXs). P22 (phox) combined with NOX to form a complex is one of the important sources of reactive oxygen species (ROS) in cells and is associated with a variety of diseases, such as cardiovascular and cerebrovascular [31]. The presence of NOX4 complex (NOX4 and p22 (phox)) in chondrocytes has been demonstrated and may play a role in chondrocyte formation or differentiation. Increased expression of p22 (phox) and NOX4 also promotes ROS production and IL-1*β* synthesis, which in turn affects OA development by mediating MMPs and IL-1*β* autocrine in chondrocytes [32]. Research shows that p22 (phox) inhibitors produce anti-inflammatory effects on isolated synovial cells and prevent ROS-mediated inflammation in human cells [33]. Age-related mitochondrial dysfunction is associated with an increase in mitochondria-driven ROS. Excessive ROS production by chondrocytes includes superoxide, hydrogen peroxide, and nitric oxide, which can lead not only to oxidative damage but also to disruption of the AKT and MAP kinase cellular signaling pathways of redox [34]. Mitochondrial dysfunction, increased production of ROS and defective scavenging of reactive oxygen species in synovial cells, activating the NF-KB signaling pathway to induce the release of pro-inflammatory cytokines (e.g., IL-1*β* and IL-6), may affect the development and progression of OA [35, 36]. ROS produced by mitochondria plays a key role in activating cellular signaling pathways to promote cell death, e.g., H_2_O_2_ produced by mitochondria can activate the MKK3/6-p38 signaling pathway to induce death of human chondrocytes [37]. GSEA analysis showed that natural killer cell cytotoxicity was involved in the progression of OA. In our study, CYBA expression was increased in OA samples and may affect the progression of OA by causing cell death through the generation of excess ROS.

SLC19A2, thiamine transporter protein (THTR) 1, transports thiamine into the cell for its efficient utilization. Thiamin is a key cofactor involved in the maintenance of carbohydrate metabolism and is involved in a variety of cellular metabolic processes in the cytoplasm, mitochondria, and peroxisomes [38]. Thiamin deficiency affects the neurological, cardiovascular, respiratory, gastrointestinal, and musculoskeletal systems [39]. Thiamin, a key molecule in glycolysis and TCA cycle, exists as thiamin pyrophosphate and plays an important role in cellular energy production [40]. In thiamin-deficient states, it limits the circulation of Krebs cycle, leading to reduced ATP synthesis, oxidative damage, and cell death. Currently, there are relatively few studies on thiamin in articular cartilage, and our study shows that SLC19A2 expression is reduced in OA samples, possibly by affecting ATP production, which in turn promotes OA progression.

Mitochondria have a complex energy-dependent feedback mechanism mediated by Uncoupling proteins (UCPs), which are conserved family proteins located in the inner membrane of mitochondria. UCPs could make proton reflux and slightly uncouple oxidative phosphorylation, thus reducing the generation rate of free radicals in mitochondria, playing the role of antioxidant, and alleviating oxidative stress. When excess ROS or ROS byproducts are produced, UCP2 is activated to induce proton leakage, thereby negatively regulating mitochondrial ROS production [41–43]. Acute activation of UCP2 by ROS can directly regulate the glutathione state of UCP to reduce ROS release and participate in cell signaling mechanisms [41]. UCP2 overexpression also prevents overproduction of superoxide and oxidative damage in pathological conditions such as stroke and ischemia/reperfusion [43]. Knocking down UCP2 in the presence of FasL or CHX + FasL increased ROS production in MSCs [44]. In addition, increasing UCP2 expression helps maintain the chondrocyte phenotype and improve mitochondrial function by reducing the production of reactive oxygen species production [45]. In patients with OA, UCP2 is upregulated by ROS activation, which plays a negative feedback role to alleviates oxidative stress damage of cells.

In summary, this study identified four hub genes (ACADL, CYBA, SLC19A2, and UCP2) associated with mitochondrial dysfunction and validated their expression in chondrocytes, which may enhance our understanding of the progression of osteoarthritis and provide prognostic biomarkers as well as therapeutic targets. At the same time, there are some limitations to our study. First, the sample size in the original dataset used in this study is relatively small and should be further examined using larger samples. Second, we only performed *in vitro* experiments in chondrocytes to verify the expression of these four genes, and further *in vivo* experimental approaches, such as knockout mice and gene overexpression, are needed to explore the effects of these four genes on OA.

## 5. Conclusions

Bioinformatics and experimental data showed that ACADL, CYBA, SLC19A2, and UCP2 were hub MDFGs in OA compared to controls. Our study may provide novel hub mitochondrial dysfunction genes with potential clinical applications for the pathology, diagnosis, and treatment of OA.

## Figures and Tables

**Table 1:** Hub genes and their primer sets.

Hub genes	Primer
ACADL	F—TGCAATAGCAATGACAGAGCC
	R—CGCAACTACAATCACAACATCAC

CYBA	F—ACCAGGAATTACTATGTTCGGGC
	R—TAGGTAGATGCCGCTCGCAATG

SLC19A2	F—TTGCCACAGACTACCTCCGT
	R—GCACTTCGACAGTAACTTGTGA

UCP2	F—GGAGGTGGTCGGAGATACCAA
	R—ACAATGGCATTACGAGCAACAT

GAPDH	F—GTCTCCTCTGACTTCAACAGCG
	R—ACCACCCTGTTGCTGTAGCCAA

## Data Availability

The data used to support the findings of this study are available from the corresponding author upon request.
